# Short-Term Changes in Quality of Life in Patients with Advanced Lung Cancer during In-Hospital Exercise Training and Chemotherapy Treatment: A Randomized Controlled Trial

**DOI:** 10.3390/jcm10081761

**Published:** 2021-04-18

**Authors:** Anna Rutkowska, Sebastian Rutkowski, Adam Wrzeciono, Oliver Czech, Jan Szczegielniak, Dariusz Jastrzębski

**Affiliations:** 1 Faculty of Physical Education and Physiotherapy, Opole University of Technology, 45-758 Opole, Poland; a.rutkowska@po.edu.pl (A.R.); j.szczegielniak@po.edu.pl (J.S.); 2 Descartes’ Error Student Research Association, Faculty of Physical Education and Physiotherapy, Opole University of Technology, 45-758 Opole, Poland; adam.wrzeciono@student.po.edu.pl (A.W.); oliver.czech@student.po.edu.pl (O.C.); 3 Department of Lung Diseases and Tuberculosis, Faculty of Medical Sciences in Zabrze, Medical University of Silesia, 41-800 Zabrze, Poland; djastrzebski@sum.edu.pl

**Keywords:** lung cancer, quality of life, QoL, pulmonary rehabilitation, NSCLC, exercise training

## Abstract

The aim of this study was to assess the impact of exercise training on the quality of life (QoL) of patients diagnosed with stage IIIB and stage IV non–small cell lung cancer (NSCLC) compared to a passive control group (CG). The exercise-trained group (ETG) consisted of 18 patients, and the CG consisted of 8 patients. The training program in the ETG consisted of two 2-week running cycles interspersed with consecutive rounds of chemotherapy with cytostatic drugs. A comparison of the changes in the Short Form (36) Health Survey (SF-36), St. George’s Respiratory Questionnaire (SGRQ), and the Functional Assessment of Cancer Therapy-Lung (FACT-L) was the primary outcome. Analysis of the results of the SGRQ and the SF-36 questionnaire did not reveal any statistically significant differences in the assessment of QoL between the examined groups. The analysis of FACT-L questionnaires showed statistically significant changes, indicating deterioration of QoL in domains describing physical well-being in the CG. Therefore, the analysis of the results of the QoL assessment did not show any significant improvements in the group of patients undergoing comprehensive exercise training, although deterioration of QoL was noted in the CG.

## 1. Introduction

Cancer is currently the second leading cause of death and disability after cardiovascular disease, responsible for around 270,000 deaths annually in Europe [1]. Morbidity and mortality statistics vary widely across the globe. About 16% of lung cancers are diagnosed during early stages; therefore, the 5-year survival rate is only 15% [2,3,4]. According to Global Cancer Observatory 2018 estimates, lung cancer is the most commonly diagnosed malignant neoplasm (2.1 million new cases, which corresponded to 11.6% of all cancer cases in 2018) [5]. Poland has some of the highest mortality and morbidity rates. Each year, around 20,000 people die because of lung cancer. Most patients (approximately 80%) have distant metastases at diagnosis [6]. Cigarette smoking or tobacco smoking is the most important risk factor for developing lung cancer. Smoking causes over 90% of lung cancer-related deaths in men and 70% in women [7].

Lung cancer patients experience a variety of distressing symptoms, many of which begin before diagnosis and continue throughout the course of the disease and its treatment, adversely affecting functional status and quality of life (QoL). The vast majority of patients, especially those with advanced disease, cannot be cured; therefore, the goal of therapy in such patients is to prolong survival and to improve QoL. Each patient’s QoL is multi-dimensional; it covers physical, functional, psychological, social, and spiritual domains, the consequence of which is the inclusion of QoL assessment in most types of clinical trials in oncology [8]. The QoL of lung cancer patients is determined by factors related to the course of the disease (disease stage, coexisting diseases) and treatment (surgery, chemotherapy, radiotherapy). Lung cancer patients report a lower degree of health-related quality of life (HRQoL) compared to other cancer patients. HRQoL reflects the way in which patients experience the impact of their disease and its treatment on the quality of their everyday life [9]. It has been shown that symptoms of lung cancer, such as dyspnea, pain, fatigue, muscle waste, and loss of strength significantly limit the physical activity of patients, thus translating into reduced independence in everyday activities [10]. In particular, the perceived pain, dyspnea, and feeling of fatigue at a high level also affect the deterioration of mental well-being, and they are considered to contribute to the emergence of depression in cancer patients [11].

The challenge of comprehensive medical care is the appropriate management of the symptoms of the disease through pharmacological, psychological, and physiotherapeutic support in order to improve QoL, especially in patients whose lung cancer excludes them from traditional treatment. The medical community is aware of the positive impact of pulmonary rehabilitation and exercise-based physiotherapy on improving the fitness and functioning of patients, thereby reducing emerging physical disabilities [12,13,14,15,16,17]. The use of rehabilitation is advisable in an attempt to reduce the ailments resulting from the course of lung cancer, which may lead to improving physical and mental health, thus improving the QoL of patients. Physical training plays an increasingly important role in the treatment of lung cancer. In recent years, the implementation of pulmonary rehabilitation in patients with lung cancer has gained recognition in the world of medicine. It has been shown that rehabilitation is safe and acceptable to patients; improves fitness, physical efficiency, and oxygen consumption; and reduces fatigue [14,15,17,18,19]. The reason for this is that exercise training challenges the entire oxygen transport pathway from the lungs, to the circulation, to the tissues—delivering oxygen to the cells. This influences gas exchange by increasing oxygen transport and enabling more efficient elimination of CO_2_ [20]. Thus, exercise is a very effective method for the relief of dyspnea [21], which is a significant limitation of patients in the advanced stages of lung cancer. In contrast to studies in the pre- and post-operative settings that indicate an improvement in physical performance and cardiorespiratory fitness, studies investigating the effects of physical exercise in non-operable patients with advanced lung cancer are rare [22]. Patients with advanced non-small cell lung cancer (NSCLC) cannot be cured; therefore, symptomatic treatment should prolong survival and improve QoL. On the other hand, the toxicity of cytostatics has side effects that negatively affect many aspects of functioning, including QoL [23]. Fatigue, loss of appetite, dyspnea, and pain have a significant negative impact on the assessment of HRQoL in patients with advanced NSCLC [24].

Therefore, the aim of this study was to evaluate the impact of in-hospital rehabilitation in patients diagnosed with stage IIIB and stage IV NSCLC who were disqualified from surgery and receiving regular chemotherapy. We hypothesized that the 4-week pulmonary rehabilitation program would improve the QoL of these patients.

## 2. Materials and Methods

### 2.1. Participants

**Figure 1:**
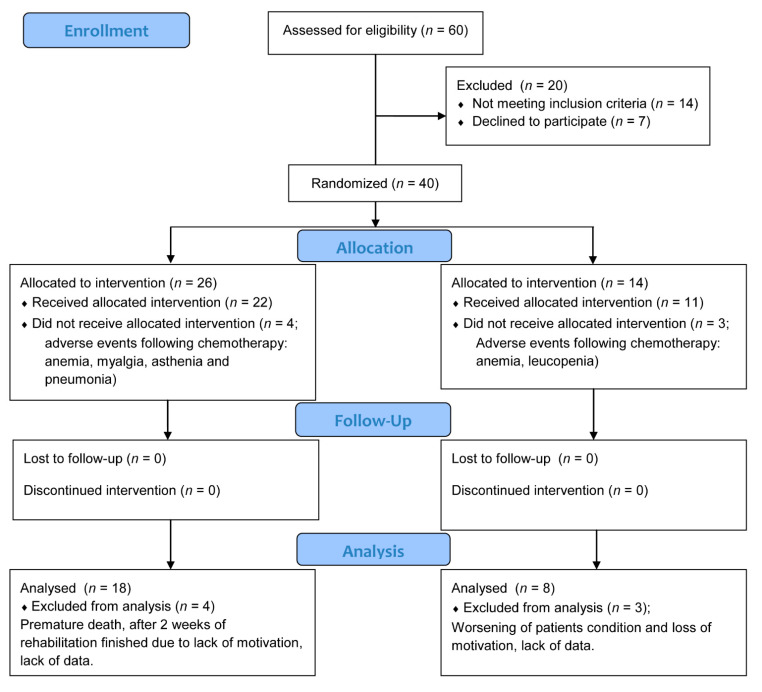
CONSORT flow diagram.

The study included 40 patients diagnosed with stage IIIB or stage IV NSCLC who were disqualified from surgery (Table 1). The research study took place at the Independent Public Clinical Hospital No. 3 of Medical University of Silesia in Katowice. It was approved by the Ethics Committee of Silesian Medical University (KNW/0022/KB1/184a/I/11/12). The research was registered in the Australian New Zealand Clinical Trials Registry: ACTRN12616001512415. The diagnosis was established within 6 weeks prior to enrollment and was confirmed by histology. In 26 patients, a full final evaluation was performed (Figure 1). The inclusion criteria were as follows: ability to perform the 6-min walk test (6MWT); Eastern Cooperative Oncology Group performance status 0–1; ability to complete the questionnaires; and willingness to participate in an exercise training program. The exclusion criteria were as follows: unstable coronary artery disease or uncontrolled hypertension; anemia (hemoglobin <10 g/dL); severe osteoarthritis; or bone or central nervous system (CNS) metastases. Patients were randomly assigned to the exercise-trained group (ETG) or the control group (CG). Randomization (ratio 2:1) was performed using Research Randomizer, a web-based service that offers instant random assignment. The randomization ratio was set to include the largest possible group of participants in the intervention due to the likelihood of improvement in health status. Sealed envelopes were used for group assignments. Twenty-six patients were assigned to the ETG, and fourteen patients were assigned to the CG. Participants in the study were assessed before and after the completion of a comprehensive rehabilitation and chemotherapy treatment program. The ETG used a 4-week stationary exercise program based on a rehabilitation program used in patients with chronic obstructive pulmonary disease (COPD), which was performed in 2-week cycles interspersed with consecutive chemotherapy infusions of cisplatidiam (80 mg/m^2^) on the first day and vinorelbine (25 mg/m^2^) on the first and eighth day (subjects in the CG received the same chemotherapy regimen). Patients participating in the study signed a consent to participate form. COPD is a group of lung conditions that cause breathing difficulties, including emphysema and chronic bronchitis. Presently, it is common to determine airway obstruction by use of a fixed cut-off point, when FEV1/FVC is less than 70%.

### 2.2. Outcomes

**Figure 2:**
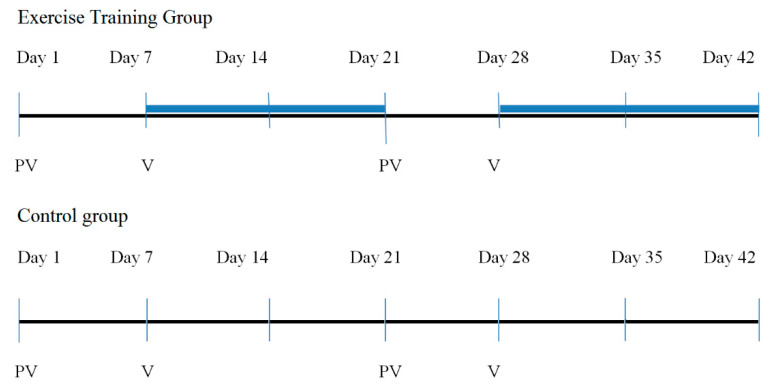
Study flow.

Comparison of the changes in St. George’s Respiratory Questionnaire (SGRQ), the Short Form (36) Health Survey (SF-36), and the Functional Assessment of Cancer Therapy-Lung (FACT-L) was the primary outcome. At baseline (day 1) and at the end of the study (day 42), QoL was assessed in both groups (Figure 2).

#### 2.2.1. St. George’s Respiratory Questionnaire

The questionnaire consists of 50 questions grouped into three subscales: symptoms, activity, and impact on life. The results of the SGRQ and its subscale are given as points between 0 and 100, with 0 points being the highest QoL and 100 points being the lowest QoL. The SGRQ is a valid measure to assess QoL in subjects with lung cancer [25].

#### 2.2.2. The Short Form (36) Health Survey

The SF-36 consists of 36 questions that cover the following eight basic topics (domains) describing health: physical functioning, physical role, bodily pain, general health, vitality, social functioning, emotional role, and mental health. Since each of the above elements contains data on both physical and mental health, answers to questions related to mental and physical health were separated from eight thematic issues, creating two separate modules for assessing health conditions: mental cumulative score and physical cumulative score. Responses obtained in the SF-36 questionnaire were converted according to the appropriate key, where the higher score result meant better health [26]. SF-36 has been validated on groups of lung cancer patients [27].

#### 2.2.3. Functional Assessment of Cancer Therapy-Lung

FACT-L assesses the functioning of patients undergoing therapy due to lung cancer. The questionnaire assesses physical well-being, emotional well-being, social well-being, functional well-being, and lung cancer subscale. The higher score result reflects the lack of restrictions or discomfort in terms of the analyzed index. This tool, also, was validated on a group of patients with lung cancer [28].

### 2.3. Interventions

In the experimental group, patients participated in two 2-week stationary exercise training camps supervised by a certified physiotherapist. Between these two periods, the patients underwent scheduled chemotherapy at the hospital. In the CG, between the initial and final assessment, the patients underwent scheduled chemotherapy in the hospital; thus, no exercise intervention was performed in this group (Figure 2).

The patients that qualified for supervised in-hospital exercise training were assigned to different intensities of the individual components of the training on the basis of initial 6MWT and spirometry assessments. Patients participated in training sessions five times a week. The training consisted of (1) 30 min of fitness and respiratory exercises; (2) 30 min of respiratory exercises focused on strengthening exercises of the diaphragm with resistance, exercises to increase costal or chest breathing, and prolonged exhalation exercise; (3) training on a cycle ergometer or treadmill for 20 to 30 min at an intensity of 30% to 80% of peak work rate according to individual tolerance [29]; (4) weighted exercise with intensity 40% to 70% of the 1 repetition maximum (1RM); (5) Nordic walking for 45 min (depending on weather and health condition of the patient); and (6) 20 min Schultz autogenic training. Heart rate and oxygen saturation were monitored continuously throughout the sessions by pulse oximetry.

Exercise training sessions were withheld in the following situations related to cancer chemotherapy: 24 h after chemotherapy; anemia (hemoglobin < 8 g/L); neutropenia (white blood cell count < 0.5 × 109 cells/μL); thrombocytopenia (platelet count < 50 × 109 μL); and participants complaining of nausea, vomiting, fatigue, disturbances of orientation, visual disturbances, weakness, muscle pain, or bone pain within the preceding 24 h.

### 2.4. Statistical Analysis

All statistical analyses were performed using Statistica 13.1 software (StatSoft, Cracow, Poland). The statistical significance level was set at α = 0.05. Continuous variables were presented as the median and interquartile range [IQR], where deemed appropriate according to the Kolmogorov–Smirnov normality test. The baseline characteristics of the groups were compared using the Mann-Whitney U test. Differences between variables within groups were compared using the Wilcoxon signed-rank test, while the differences of posttest values minus pretest values (Δ Post-Pre) were assessed with the Mann-Whitney U test. The size of the between-group effects was determined by Morris effect size d [30] and classified as follows: 0.1–0.3, small effect; 0.3–0.5, intermediate effect; and ≥0.5, strong effect [31]. The sample size was calculated on the basis of the previous meta-analysis according to the quality of life in patients with lung cancer [32]. We used G*power 3.1.9 software to calculate the sample size. The calculation was based on the t-test (Wilcoxon signed-rank test (matched pairs)). The type I error rate was set at 5% (alpha-level 0.05). We assumed the effect size at 0.64 based on the previous meta-analysis; the type II error rate gave 99% power. The appropriate minimum sample size for this study was 40 subjects.

## 3. Results

The characteristics of the ETG were closely matched to those of the CG group in terms of gender, age, weight, and height (Table 1). As shown in the CONSORT chart (Figure 1), of the 26 patients randomized to the ETG, 18 completed a 4-week training program and passed the final evaluation. Of the 14 participants enrolled in the CG, 8 completed the study and passed the final evaluation. Four participants in the ETG and three participants in the CG did not complete the study because of chemotherapy events (due to anemia, muscle pain, asthenia, pneumonia, or renal failure) or failure to complete all questionnaires. No adverse events were reported in the ETG.

The analysis of the SGRQ in the ETG and the CG did not reveal any statistically significant changes. However, the intermediate effect size was noted in the symptoms domain (d = −0.31) and the impact of life domain (d = −0.43), which suggests relatively better results in the ETG (Table 2).

The analysis of the results of the SF-36 questionnaire showed non-significant changes in both groups in all analyzed domains. However, the intermediate effect size was noted in the vitality domain (d = 0.506) and the social functioning domain (d = 0.424) (Table 3). 

In the experimental group, the analysis of the FACT-L questionnaire did not reveal any statistically significant changes. However, in the CG, statistically significant deterioration in physical well-being was noted (*p* < 0.02), with strong effect size (d = 0.753). The analysis of the social and emotional well-being domains, and likewise, the lung cancer symptoms domains between groups showed intermediate effect size (Table 4).

## 4. Discussion

This study assessed the effect of comprehensive physiotherapy in patients with stage IIIB and stage IV lung cancer that were disqualified from surgery and undergoing chemotherapy. The FACT-L, SF-36, and SGRQ were used to assess QoL. The statistical analysis of the results did not confirm the established hypothesis assuming an improvement in the QoL of patients undergoing 4-week pulmonary rehabilitation. The mean values of the examined parameters indicated a slight influence manifested in the improvement in QoL in individual domains of the questionnaires in the experimental group in comparison to the CG. In the CG, there was a large deterioration in QoL in the analyzed parameters. Furthermore, small to large effect sizes were noted between groups. Thus, this might suggest that the practical conclusions of the study include maintenance of QoL in the study group while QoL worsened in the control group—those not undergoing rehabilitation.

Our results are in line with Henke et al., who investigated patients with advanced NSCLC that underwent comprehensive rehabilitation with components of endurance, fitness, and muscle strength training during cytostatic treatment [33]. QoL was assessed using the European Platform of Cancer Research (EORTC) quality of life questionnaire (QLQ-30). The analysis of results showed a positive effect on the patients’ endurance and strength capacity; however, only single factors influencing the patients’ quality of life were improved in the group subjected to physiotherapy. Additionally, the studies by Jastrzębski et al. and Hwang et al. showed no significant changes in QoL between the CG and the group of patients with advanced lung cancer receiving physiotherapy during chemotherapy [14,34].

The lack of significant improvement in the QoL of the group of patients with inoperable NSCLC may result from the advanced stage of the disease and the transition to palliative chemotherapy. Although palliative treatment aims at improving survival and reducing symptoms, it appears that the therapy frequently focuses on medical and physiological aspects with little attention given to psychosocial components [33]. A systematic literature review by Dittus et al. showed that 60% of patients with advanced neoplasms undergoing physical training had improved QoL. However, it is known that lung cancer patients have lower QoL compared to patients with other cancers [9,35]. The long-lasting and debilitating course of the disease and treatment significantly impairs health reserves in terms of physical, social, and cognitive functioning and in the severity of the disease symptoms [36]. It has been shown that lung cancer patients are more affected by psychological stress, anxiety, and depression [37,38]. A systematic literature review by Cavalheri et al. showed that physical training in patients with NSCLC after surgery resulted in an improvement in the physical component of the overall HRQoL [39]. In a study by Brocki et al., the short- and long-term effects of supervised training were assessed in patients after lung cancer surgery. The supervised training was 10 weeks long, and the session was held once a week for 60 min. After 4 months, there was a statistically significant improvement in the pain parameter of the SF-36 questionnaire and an improvement in the mean results in the domain of physical functioning compared to the group where training was not supervised; however, after 12 months, the QoL assessment showed no significant changes. An improvement was even observed in the group without supervised training [40]. However, the results of studies of the QoL of patients with an advanced stage of the disease are divergent, and no unequivocal impact of rehabilitation on QoL has been demonstrated.

The long-term exercise programs have been shown to provide health benefits for patients. Riedl et al. investigated the change of psychological distress and HRQOL during a multidisciplinary inpatient rehabilitation on a large sample (*n* = 939) of mixed cancer survivors. Analysis of the results showed clinically meaningful improvement in almost all domains of the EORTC QLQ-C30 as well as in anxiety and depression (HADS) [41]. In a study by Li et al., comprehensive physiotherapy was initiated before surgery and lasted 6 months after lung cancer surgery, with QoL assessed 3 and 6 months after the operation. Three months after rehabilitation, the experimental group showed a statistically significant increase in individual domains describing general health, physical and emotional functioning, the performance of social roles, the level of perceived fatigue, and loss of appetite within EORTC QLQ-C30 compared to the CG. Six months after the intervention, the experimental group showed an increase in the overall functional QoL scale, and symptoms were also reduced compared to the CG. This decrease was significant on the scales of fatigue, pain, dyspnea, insomnia, loss of appetite, and constipation [42]. Similar studies were carried out by Jannsen et al., with the exception of the CG, where 12-week supervised rehabilitation after lung cancer surgery was carried out. QoL was assessed using the SF-36 and the FACT-L questionnaire. After completing the physiotherapy program, a statistically significant improvement was noted in the cumulative assessment of physical and mental health in the SF-36 questionnaire and in the FACT-L subscale for lung cancer symptoms and fatigue [12]. Comparatively, results of our 4-week study showed a non-significant statistical increase in the mean values of the SF-36 questionnaire in the domains describing pain, mental health, and mental health score assessment. However, Jannsen et al. analyzed the outcomes of patients with NSCLC stage I to IIIA, while we analyzed results of patients with advanced lung cancer (stage IIIB and IV) who received palliative chemotherapy, and this presumably explains the reported results. Therefore, even a minor improvement in the mental area can be perceived as a success. It is important to emphasize that our exercise program was mainly focused on the somatic elements of the patients; the only element interacting with each patient’s psyche was Schultz autogenic training, performed 1 × a day for 20 min. Thus, it appears that the lack of personalized psychological interventions may have contributed to the modest improvement in QoL. The solution to the lack of availability of qualified oncology psychotherapists may be the incorporation of new technologies such as virtual reality. In recent years, growing interest in supplementing standard rehabilitation with virtual reality (VR) therapy, as well as the wide application of VR in various areas of rehabilitation, have been observed [43]. Numerous studies have indicated the positive effect of VR therapy as supportive therapy for improvement of QoL [44,45], and likewise, the first studies in oncology patients have appeared [46]. Such a solution can be applied by a physiotherapist, and hence, the presence of specialists will not be required during VR psychotherapy. In our previous study, we investigated the effectiveness of using immersive virtual therapy, in comparison to Schultz autogenic training, to reduce levels of stress, anxiety, and depression in patients with COPD. The results revealed that VR therapy was a significantly more effective tool in reducing anxiety and depression symptoms in COPD patients than traditional therapy [47].

A large-sample study by Hechtner et al. investigated the QoL in 657 patients who had survived a primary NSCLC diagnosis at least 1 year and was aimed at identifying factors associated with global QoL, physical functioning, emotional functioning, fatigue, pain, and dyspnea. Compared to the age- and sex-standardized general population, clinically meaningful differences in the QoL detriment were found in almost all domains; lung cancer survivors had clinically relevant poorer global QoL. The main factor associated with poorer QoL was mental distress, and the main factor associated with better QoL in almost all primary QoL scales was higher physical activity. It was concluded that lung cancer survivors experience both functional restrictions and symptoms that persist long-term after active treatment ends [48]. These results confirm previous reports that the greatest impact of comprehensive rehabilitation in patients of all stages of the disease is manifested in the domain describing physical functioning with the use of various QoL questionnaires. The recommendations of the American College of Sports Medicine indicate that physical activity of an appropriately selected form, intensity, and duration is an inseparable component of the treatment of lung cancer patients [49].

Although this study provides insights into the QoL of patients with advanced lung cancer, we recognize that some limitations should be considered. First, due to the specificity of the patient group, the study group was small. It is difficult to gather a group of patients who will agree to be physically active during chemotherapy, but it is worth the effort because it gives patients the feeling of attention from doctors and physiotherapists and the feeling that they are not only offered medication. Second, it is possible that there could have been a bias in the reporting of quality of life status. But, because our questionnaire asked respondents to indicate the QoL for two different periods (before and after 4 weeks), we presume that the internal consistency of the respondents led them to report the same bias. Third, the follow-up assessment could provide additional valuable information on the effectiveness of exercise training. Previous studies showed that improvements achieved during the rehabilitation tend to further increase in the follow-up period. Fourth, chemotherapy may also have altered quality of life, but we were unable to objectively assess the impact of this treatment regimen. Finally, for an in-depth analysis of determinants of quality of life, levels of anxiety and depression would also need to be assessed.

## 5. Conclusions

The analysis of the results of the QoL assessment did not show any significant improvement in the group of patients undergoing comprehensive exercise training; however, significant deterioration of physical well-being in the CG was observed. It is worth noting that there was a noticeable improvement in the values describing the individual domains in the ETG. It seems that intervention programs in patients with advanced lung cancer should include personalized psychological support. Nevertheless, considering the physical benefits (fitness and capacity) associated with rehabilitation for this group of patients, the implementation of exercise training is recommended. 

## Figures and Tables

**Table 1:** Characteristics of participants completing the study.

	Exercise Training Group (*n* = 18)	Control Group (*n* = 8)	*p*
Age, mean (SD)	60.4 (7.2)	62.2 (9.0)	>0.05
Male, *n* (%)	16 (89)	8 (100)	>0.05
BMI, mean (SD)	23.9 (3.7)	22.1 (2.2)	>0.05
Comorbidties			
COPD, *n* (%)	10 (55)	4 (50)	>0.05
Coronary artery disease history, *n* (%)	3 (17)	1 (13)	>0.05
Diabetes, *n* (%)	5 (28)	3 (37)	>0.05
Smoking history			
Current	0 (0)	0 (0)	>0.05
Former	15 (83)	6 (75)	>0.05
Never	3 (17)	2 (25)	>0.05
Performance status [ECOG/WHO]			
0, *n* (%)	3 (17)	1 (12)	>0.05
1, *n* (%)	15 (83)	7 (88)	>0.05
Diagnosis			
Adenocarcinoma, *n* (%)	12 (67)	5 (62)	>0.05
Squamous cc, *n* (%)	6 (33)	3 (38)	>0.05
TNM status			
T2N2M0	14 (78)	6 (75)	>0.05
T2N2M1	4 (22)	2 (15)	>0.05
Tumor stage			
IIIB, *n* (%)	14 (78)	6 (75)	>0.05
IV, n (%)	4 (22)	2 (15)	>0.05

BMI: body mass index; COPD: chronic obstructive pulmonary disease; ECOG: Eastern Cooperative Oncology Group; TNM: tumor node metastasis; WHO: World Health Organization.

**Table 2:** Baseline and 6-week SGRQ results for the two groups.

	ETG Pre	ETG Post	p *	CG Pre	CG Post	p *	Between Group Δ Post-Pre p †	Effect Size
SYMPTOMS	48.18 [27.86–60.65]	37.53 [27.44–55.49]	<0.27	38.52 [6.65–55.64]	28.36 [9.94–56.06]	<0.75	0.66	−0.31
ACTIVITY	32.33 [11.21–53.53]	38.30 [17.12–53.49]	<0.82	23.63 [11.87–41.42]	29.93 [20.33–50.40]	<0.73	0.74	−0.115
IMPACT OF LIFE	20.03 [8.21–41.00]	17.43 [10.93–21.22]	<0.39	20.56 [9.92–25.65]	17.25 [11.42–21.76]	<0.61	0.30	−0.43
GLOBAL	24.61 [14.91–49.19]	25.54 [17.15–31.36]	<0.35	26.08 [11.22–35.85]	24.80 [10.45–30.41]	<0.50	0.50	−0.171

*—within-group analysis (Wilcoxon signed-rank test); †—between-group analysis (Mann-Whitney U test).

**Table 3:** Baseline and 6-week SF-36 results for the two groups.

	ETG Pre	ETG Post	*p* *	CG Pre	CG Post	*p* *	Between Group Δ Post-Pre *p* †	Effect Size
PF	47.75 [36.20–50.90]	47.75 [38.10–52.90]	<0.36	52.80 [43.35–55.95]	52.25 [37.05–54.80]	<0.07	0.15	0.199
RP	43.40 [28.00–56.20]	42.10 [28.00–49.20]	<0.22	43.40 [39.75–56.90]	47.10 [39.75–48.30]	<0.34	0.85	−0.123
BP	39.65 [33.60–62.10]	45.80 [37.50–51.60]	<0.48	53.25 [41.80–62.10]	56.60 [48.20–62.10]	<0.28	0.93	−0.074
GH	41.50 [33.60–46.20]	41.05 [38.20–42.90]	<0.55	43.40 [37.70–48.20]	48.20 [38.90–51.75]	<0.55	0.93	−0.059
VT	51.65 [34.90–60.90]	49.50 [42.00–58.50]	<0.81	55.20 [48.95–64.55]	58.30 [34.95–61.45]	<0.26	0.22	0.506
SF	43.40 [30.00–51.10]	41.75 [35.40–51.40]	<0.87	54.10 [43.20–56.80]	51.40 [35.05–56.80]	<0.06	0.34	0.424
RE	55.30 [40.30–55.30]	49.15 [23.70–55.30]	<0.44	46.15 [36.45–55.90]	44.20 [44.20–53.95]	<0.89	0.93	−0.094
MH	43.75 [34.50–52.70]	47.70 [39.10–50.00]	<0.58	48.60 [38.75–57.05]	48.60 [34.50–57.05]	<0.73	0.49	0.239
PCS	41.85 [35.30–48.90]	42.85 [35.80–47.80]	<0.66	51.25 [42.55–54.05]	50.55 [39.40–53.20]	<0.36	0.22	0.179
MCS	46.70 [40.70–52.30]	47.25 [38.10–53.10]	<0.56	48.20 [42.15–57.80]	47.25 [37.95–59.20]	<0.57	0.91	0.111

PF: Physical functioning; RP: role physical; BP: bodily pain; GH: general health; VT: vitality; SF: social functioning; RE: role emotional; MH: mental health; PSC: physical health score; MCS: mental health score; *—within-group analysis (Wilcoxon signed-rank test); †—between-group analysis (Mann-Whitney U test).

**Table 4:** Baseline and 6-week FACT-L results for the two groups.

	ETG Pre	ETG Post	*p* *	CG Pre	CG Post	*p* *	Between Group Δ Post-Pre *p* †	Effect Size
PWB	20.5 [13.0–23.0]	18.5 [15.0–24.0]	<0.84	25.5 [22.0–28.0]	19.5 [16.5–25.0]	<0.02	0.02	0.753
SWB	22.5 [18.0–25.0]	20.0 [18.0–24.0]	<0.40	25.5 [22.0–28.0]	19.5 [16.5- 25.0]	<0.93	0.64	−0.459
EWB	17.0 [8.0–22.0]	16.5 [12.0–21.0]	<0.94	24.5 [19.0- 25.5]	23.5 [18.5–25.5]	<0.34	0.50	−0.524
FWB	18.5 [16.0- 21.0]	19.5 [15.0- 24.0]	<0.56	17.5 [15.5–19.5]	19.0 [15.5–22.0]	<0.44	0.78	−0.251
LC	17.5 [16.0–24.0]	22.5 [18.0–24.0]	<0.17	17.5 [15.5–22.0]	22.0 [15.5–5.0]	<0.11	0.10	0.592
FACT-L	91.0 [76.0–113.0]	92.5 [77.0–108.0]	<0.87	99.5 [95.5–118.0]	106.0 [84.5–115.0]	<0.22	0.49	0.058

PWB: physical well-being; SWB: social well-being; EWB: emotional well-being; FWB: functional well-being; LC: lung cancer; FACT-L: Functional Assessment of Cancer Therapy-Lung; *—within-group analysis (Wilcoxon signed-rank test); †—between-group analysis (Mann-Whitney U test).

## Data Availability

The data presented in this study are available on request from the corresponding author.
